# Attenuated UV Radiation Alters Volatile Profile in Cabernet Sauvignon Grapes under Field Conditions

**DOI:** 10.3390/molecules200916946

**Published:** 2015-09-17

**Authors:** Di Liu, Yuan Gao, Xiao-Xi Li, Zheng Li, Qiu-Hong Pan

**Affiliations:** 1 Center for Viticulture & Enology, College of Food Science & Nutritional Engineering, China Agricultural University, Beijing 100083, China; E-Mails: liudi19903@126.com (D.L.); gaoyuan060117@163.com (Y.G.); lixxcau@gmail.com (X.-X.L.); 2 Food Science and Human Nutrition Department, Institute of Food and Agricultural Sciences, University of Florida, Gainesville, FL 32611, USA; E-Mail: Jameslee0221@ufl.edu

**Keywords:** UV attenuation, volatile compounds, grape berry

## Abstract

This study aimed to explore the effect of attenuated UV radiation around grape clusters on the volatile profile of Cabernet Sauvignon grapes (*Vitis vinifera* L. cv.) under field conditions. Grape bunches were wrapped with two types of polyester films that cut off 89% (film A) and 99% (film B) invisible sunlight of less than 380 nm wavelength, respectively. Solar UV radiation reaching the grape berry surface was largely attenuated, and an increase in the concentrations of amino acid-derived benzenoid volatiles and fatty acid-derived esters was observed in the ripening grapes. Meanwhile, the attenuated UV radiation significantly reduced the concentrations of fatty acid-derived aldehydes and alcohols and isoprenoid-derived norisoprenoids. No significant impact was observed for terpenes. In most case, these positive or negative effects were stage-dependent. Reducing UV radiation from the onset of veraison to grape harvest, compared to the other stages, caused a larger alteration in the grape volatile profile. Partial Least Square Discriminant Analysis (PLS-DA) revealed that (*E*)-2-hexenal, 4-methyl benzaldehyde, 2-butoxyethyl acetate, (*E*)-2-heptenal, styrene, α*-*phenylethanol, and (*Z*)-3-hexen-1-ol acetate were affected most significantly by the attenuated UV radiation.

## 1. Introduction

**Figure 1:**
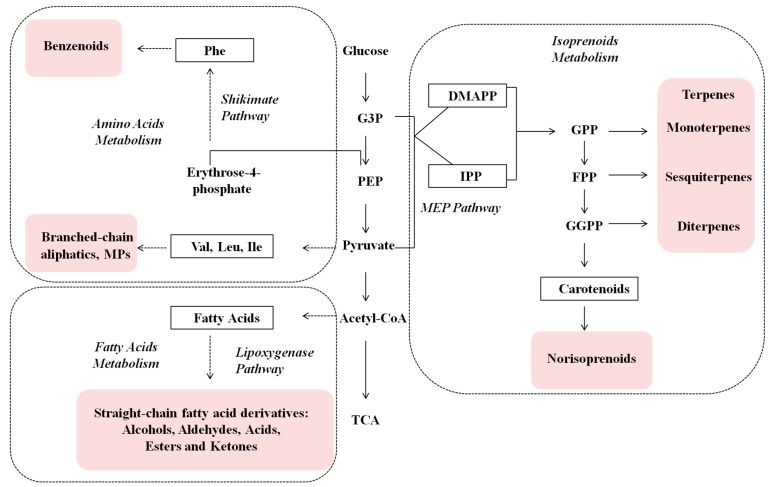
Biosynthetic pathways of three classes of volatile compounds in grapes. Metabolism and pathway names are italicized, whereas volatiles are in pink shadow. Abbreviations: G3P, glyceraldehyde 3-phosphate; PEP, phosphoenolpyruvic acid; Acetyl-CoA, acetyl coenzyme-A; TCA, tricarboxylic acid cycle; DMAPP, dimethylallyl pyrophosphate; IPP, isopentenyl pyrophosphate; GPP, geranyl pyrophosphate; FPP, farnesyl pyrophosphate; GGPP, geranylgeranyl pyrophosphate; Phe, l-phenylalanine; Val, valine; Leu, leucine; Ile, isoleucine.

Volatile compounds are naturally produced in plants. At present, a total of 1700 volatile components have been identified from more than 90 plant species [1]. Based on their biosynthetic pathways, volatiles in grapes are commonly divided into three major classes: isoprenoid derivatives, amino acid derivatives, and fatty acid derivatives (Figure 1). These three classes of volatiles have different contributions to grape and wine aroma quality.

Terpenoids are biologically synthesized from isopentyl pyrophosphate (IPP) and dimethylallyl pyrophosphate (DMAPP). These precursors are formed from three molecules of acetyl-CoA through cytosolic mevalonic-acid (MVA) pathway [2] or pyruvate and glyceraldehyde-3-phosphate under plastidial 2-C-methylerythritol-4-phosphate (MEP) pathway [3]. Terpene profile, especially monoterpene profile, in muscat-type and aroma-type grapes, primarily determines the aroma attributes of respective wines [4]. Norisoprenoids are yielded from the degradation of carotenoids that are biosynthesized from IPP. β*-*Damascenone and ionone are two important C13-norisoprenoid components and their levels are above their thresholds in grapes. Therefore, they play a primary role in contributing floral odor to many grape varieties, such as Cabernet Sauvignon [4].

Amino acid-derived volatiles include benzenoids, branched-chain aliphatics, and methoxypyrazines. Benzenoids are a group of aromatic volatile components derived from l-phenylalanine, a substrate synthesized via shikimate pathway [4]. Branched-chain aliphatic volatiles, including their aldehydes, alcohols, and esters, are mainly synthesized from valine, leucine and isoleucine. Methoxypyrazines (MPs) are nitrogenated heterocyclic products of leucine and isoleucine metabolisms [5]. MPs have extremely low sensorial thresholds and are commonly described as bell peppers or green leaf odor [6]. MPs exist at detectable levels only in Sauvignon Blanc, Cabernet Sauvignon, and Merlot, and they are important contributors to the distinctive vegetative characters of these varieties [7].

Straight-chain alcohols, aldehydes, esters, acids, and ketones are basically originated from fatty acids via α*-* or β*-*oxidation or lipoxygenase (LOX) pathway [8]. In grapes, aliphatic volatiles predominantly include short-chain aldehydes and alcohols [9], most of which are described as “green” aromas [10]. These aldehydes and alcohols can be converted into the corresponding acetate esters during wine making and aging processes, which further affects wine aroma quality.

Ultraviolet light (UV) is considered one of several major environment factors that affect the development and physiology of plants. A number of studies have been conducted to investigate the impact of enhancing or reducing UV radiation on plant secondary mechanism. For example, the synthesis of flavonoids has been proved as a class of important protective compounds against UV-stimuli. Of these investigations, the UV-B (280–315 nm) effects have been the focus of much attention. This is because only a certain proportion of UV-B can pass through the ozone layer of earth, and its intensity under natural conditions is significantly impacted by regions and climates [11,12]. In contrast, almost all UV-A (315–390 nm) can reach the earth surface, whereas UV-C (≤280 nm) is almost absorbed by the ozone layer. In recent years, the intensity of UV radiation reaching the earth surface has increased with a continuous depletion of atmospheric ozone [13,14]. The alteration of flavor metabolism in grapes in response to enhancement of UV radiation has attracted great interest [15,16]. Excessive UV radiation has been reported to promote the accumulation of phenolics and carotenoids in grapes [15,16,17]. Our previous results also suggested that supplemental UV-A, UV-B, and UV-C radiation differentially improved the biosynthesis of anthocyanins and flavan-3-ols in grapes *in vitro* [18,19]. Some studies also reported the modifications of volatile profile, such as terpenes [20,21,22], C13-norisoprenoids [20,23], and isobutyl methoxypyrazine (IBMP) [24] of grape berries and wines after applying different intensities of UV-radiation to grapes. In general, enhancing UV radiation increased the level of terpenes [20,25,26,27]. However, reduced UV radiation treatment showed inconsistent results. For example, some investigations indicated that UV-B radiation reduction down-regulated the accumulation of terpenes in Grindelia chiloensis (Asteraceae) and Malbec grapes [22,28]. Similarly, the concentration of terpenes was lower in Sauvignon Blanc wine made of the grapes covered by UV radiation-reducing sheets in field [21]. However, Song *et al*., (2015) reported that blocked UV radiation treated Pinot Noir grape wine showed the similar level of terpenes compared to wine made of normal grapes [20]. The level of isobutyl methoxypyrazine in Sauvignon Blanc grapes under attenuated UV radiation was as similar as that in normally grown grapes [24]. β*-*Damascenone level was higher in Riesling wine made of UV exclusion-treated grapevines [23], whereas the concentration of the other C13-norisoprenoids was not significantly altered [20]. These results suggested that reduced UV-radiation effect on volatile metabolisms might be dependent on variety and cultivation differences. It should be also noted that these previous studies only focused on some specific volatile compounds in grapes and wines.

Most of these supplemental UV radiation effect studies were conducted in growth chambers or greenhouses [25,29] using isolated fruits [26,27]. Only a few field experiments have been studied using lessened UV radiation with polyester films [20,21,30]. In this study, a field study was carried out with two types of filter films that removed much of the radiation at shorter wavelength (≤380 nm). The objectives of the study were to examine the response of grape flavor metabolism to attenuated UV radiation and the stage-dependence of this response. The effect of attenuated UV radiation on the formation of volatiles during the grape development stages was assessed in terms of their biosynthetic pathways. The results of this study could provide the guidance to viticulturists and winemakers. From their perspectives, it is essential to identify the viticulture practices that can improve grape and wine aroma quality since some cultivation measurements, such as plastic greenhouse, rain shelter cultivation and fruit bagging, could result in the reduction of solar UV radiation.

## 2. Results and Discussion

### 2.1. Effect of Attenuated UV Radiation on Three Major Classes of Volatiles

**Figure 2:**
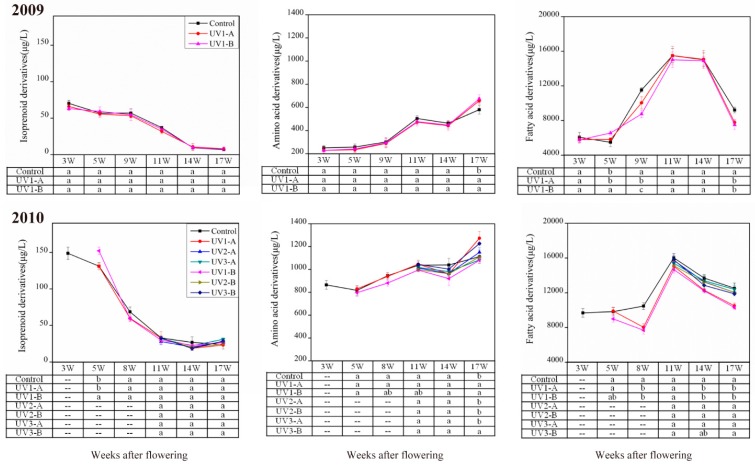
Effect of the attenuated UV radiation on the concentration of three major classes of volatiles in developing “Cabernet Sauvignon” grapes. The abbreviation of UV treatments are described in the “Experimental section”. Data are means ± SE of three replicates (*n* = 3). At the same development stage, different letters represent significant difference among treatments (*p* ≤ 0.05).

Volatile compounds are divided into three classes according to their biosynthetic pathways. These three classes of volatiles showed the similar variation trends in the developing grapes under these two experimental vintages. However, great differences were observed in their concentration (Figure 2). For example, the concentrations of amino acid-derived and isoprenoid-derived volatiles in the 2010 vintage were almost two or even more times higher than those in 2009. Moreover, a total of 102 volatile compounds were identified in the 2010 vintage, whereas only 72 volatiles were present in the 2009 vintage (Supplementary Table S1). The difference in the composition and concentration of volatiles between these two year vintages might result from the distribution of rainfall amount during the growing season. Meteorological record from the local meteorological station showed that the accumulative rainfall from June to September was 226.6 mm and 273.9 mm in 2009 and 2010, respectively. The rainfall mainly occurred in July, 2009, with a rainfall amount of 112.4 mm. However, in 2010 the monthly rainfall amount was relatively homogeneous. It was also observed that the sunshine hours, average air temperature, and average daily temperature difference were similar between these two vintages. Except for benzenoids, no differences were observed in the composition of the other volatiles in the same vintage.

Isoprenoid-derived volatiles tended to decline during grape development in both vintages, and the lowest level was observed at harvest stage for both treated grapes and the control in 2009, but in 2010, their total concentration in the treated grapes slightly increased at harvest. Except that the UV1-B- treated grapes at 5 waf in 2010 showed higher concentration of isoprenoid-derived volatiles than the control (Figure 2), no significant differences were observed between the attenuated UV-treated grapes and the control in the other stages across the two vintages. This implied that the impact of attenuated UV radiation on the isoprenoid metabolism in grape berries could be limited.

The concentration of amino acid-derived volatile compounds increased with the berry maturation, except for a slight decline at 14 waf. At harvest, the attenuated UV radiation treatment with both polyester film A and B significantly enhanced the accumulation of amino acid-derived volatiles in these two vintages. The UV3-B treated grapes (attenuated UV from the onset of veraison to harvest) contained greater level of amino acid-derived volatiles compared to the control (Figure 2). These results indicated that the attenuated UV radiation intensity and treatment stages play important roles in affecting the metabolisms of amino acid-derived volatiles in grapes.

Fatty acid-derived volatile compounds accounted for over 80% of the total concentration. Overall, it showed an increasing trend, and then a decrease in the development stages of grapes. Apart from the grapes at 5 waf in 2009, the attenuated UV-radiation treated grapes had lower level of fatty acid-derived volatiles compared to the control. Particularly, the UV1 treatments with both polyester film A and B reduced the accumulation of these volatiles in both of the vintages (Figure 2). This indicated that reducing UV radiation might down-regulate the synthesis of fatty acid derivatives. The UV1-B treated grapes displayed the most obvious difference in accumulation of amino acid-derived and fatty acid-derived volatiles, followed by UV1-A treated grapes and then the control. This indicated that UV radiation influence was dose-dependent.

The above analyses indicated that the effects of the attenuated UV1 radiation were similar between these two year vintages. Moreover, the attenuated UV radiation treatments with film A and B also showed similar effects on the alteration of volatile metabolisms. Therefore, the results in the 2010 vintage were further selected to investigate whether the effect of attenuated UV radiation altered metabolic pathways during grape development and to elucidate whether the attenuated UV-radiation applications were also dependent on development stages.

#### 2.1.1. Effect on Isoprenoid Volatile Compounds

**Figure 3:**
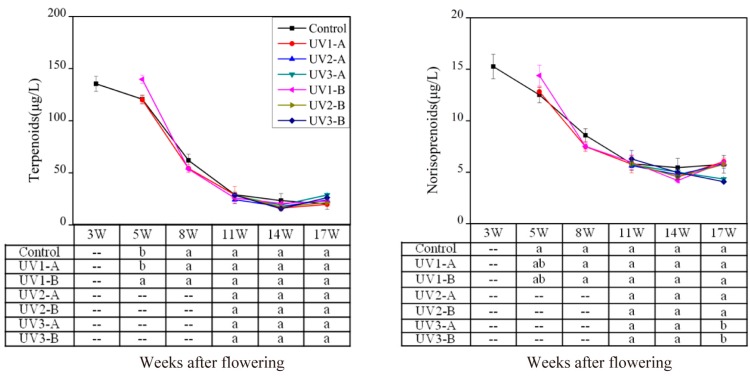
Effect of the attenuated UV radiation on the concentration of terpenes and norisoprenoids in developing “Cabernet Sauvignon” grapes. For more information, please refer to the explanation in Figure 2.

Isoprenoid metabolism yields terpenes and norisoprenoids (Figure 1). Terpenes are dominant volatiles in the family of isoprenoid derivatives, and determine the evolutionary trend of this volatile class. In this study, 18 terpene compounds were identified (Supplementary Table S1), and their total concentration had a decreasing trend throughout the development stages (Figure 3). The similar variation pattern was also reported previously in Cabernet Sauvignon cultivar [9]. Except that the UV1-B treated grapes showed higher level of terpenes at 5 waf, no significant differences were observed between the control and the treated grapes in any other stages (Figure 3). This suggested that UV radiation attenuation exerted an enhancing effect before veraison but did not impact the level of terpenes at harvest. Similarly, Song *et al*. (2015) found that terpene level in UV-exclusion-treated Pinot Noir grape wine had no significant difference compared to the control [20]. However, Šuklje *et al*. (2014) [21] observed that the content of terpenes was significantly lower in attenuated UV-radiation Sauvignon Blanc grape wine. These indicated that cultivar differences might affect UV radiation effect. Most of the terpene compounds are described as fruity-flowery aroma. In neutral varieties like “Cabernet Sauvignon”, terpenes are usually at very low levels with a minimal flavor impact [31].

Eight norisoprenoids were identified in this study, including β*-*damascenone, β-ionone, geranylacetone, 5-hepten-2-one,6-methyl-, *cis-*theaspirane, nerylactone, TCH (Cyclohexanone, 2,2,6-trimethyl-), and dihydroedulan I (Supplementary Table S1). During the development stages, the concentration of norisoprenoids continuously decreased in the UV3-A and the UV3-B treated grape berries. However, these volatile levels in the other UV treated grapes and the control showed a decrease during the development stages, but then a slight increase at harvest. The norisoprenoid levels in the UV3-A and the UV3-B treated grapes were lower than the control at harvest (Figure 3). Norisoprenoids in grapes are yielded by photochemical and/or enzymatic degradation of carotenoids in the skin and pulp [32]. The degradation of carotenoids has been reported to be inhibited in grapes under low UV-B radiation [15,16]. As a result, the concentration of carotenoids at harvest was higher compared to that under the ambient UV-B radiation. In the present study, the reduction of norisoprenoids in the UV3 treated grapes might result from the inhibition of carotenoid degradation in the grapes exposed to low UV-B radiation. It should be noted that no significant changes in the norisoprenoid concentration were observed between the UV1 treated and the control grapes even though the UV1 treatment window (from 3 waf to 17 waf) covered the UV3 treatment period. This might be because the most dramatic changes in the composition of grape berries normally occur during veraison or ripening phase. During that time, sugar/acid balance and yields of flavor and aromatic compounds and their precursors play primary roles in determining the flavor attributes of grapes [33]. Therefore, we speculated that compared with the attenuated UV radiation beginning from 3 waf (for example UV1 treatments), the UV radiation attenuation from the onset of ripening (for example UV3 treatment) could cause a stronger response to grapes under the sudden UV stress at this specific time point. In addition to norisoprenoids, some of volatiles mentioned below also displayed more significant modifications in the UV3 treated grapes compared to the UV1 treated grapes.

#### 2.1.2. Effect on Amino Acid-Derived Volatiles

**Figure 4:**
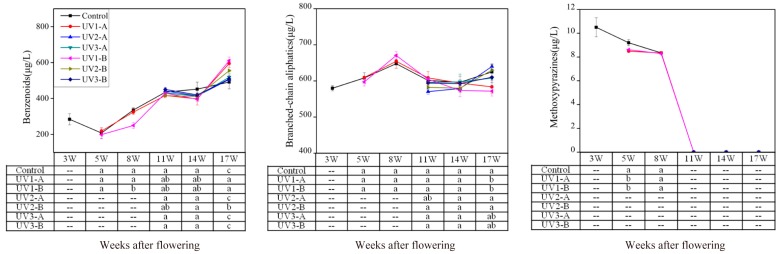
Effect of attenuated UV radiation n the concentration of amino acid-derived volatiles in developing “Cabernet Sauvignon” grapes. For more information, please refer to the explanation in Figure 2.

Amino acid-derived volatiles include benzenoids, branch-chain aliphatics, and methoxypyrazines (Figure 1). Phenylalanine is formed from shikimate acid pathway and further metabolized to yield benzenoids. Twenty benzenoids were identified in the 2010 vintage grapes (Supplementary Table S1). Benzoic acid ethyl ester, naphthalene, and 2-methoxy-phenol were only detected in the grapes at harvest. The total concentration of benzenoids showed an increasing trend during the berry maturation, and reached the highest level at harvest (Figure 4). The similar pattern was also reported by Kalua *et al*. (2009) in Cabernet Sauvignon grapes [9].

Although the concentration of benzenoids in the UV1-B treated grapes at 8-waf was lower than the control, all the UV attenuation treatments resulted in an enhancement in the concentration of benzenoids at harvest. The UV1-A, the UV1-B (UV attenuation from fruit-set to the harvest), and the UV2-B treatments (UV attenuation from fruit-set to the onset of veraison) caused a more rapid accumulation of benzenoids during grape maturation, and eventually led benzenoids to a higher level at harvest compared with the control (Figure 4). The other three treatments (UV2-A, UV3-A, and UV3-B) also enhanced the benzenoid level although they were not statistically significant. The enhancement in the concentration of benzenoids was the greatest in the UV1-B treated grapes, followed by the UV1-A and the UV2-B grapes. These suggested that the formation of benzenoids might be intensified with the intensity reduction of solar UV radiation. This activation might be related to the action of benzenoid volatiles as plant defense molecules [4,34]. The shikimate pathway mediates the flow of carbon from metabolism of carbohydrates to biosynthesis of aromatic compounds in plants. It has been demonstrated that environmental stresses can activate the shikimate acid pathway and further promote the accumulation of secondary metabolites [18,35,36]. In the present study, most of the solar UV components reaching the fruits had been removed, altering the microclimate around the fruit zone and consequently stimulating the accumulation of secondary metabolites including benzenoids in the grape berries.

Branched-chain aliphatic volatiles and methoxypyrazines in grapes are derived from three branch-chain amino acids: valine, isoleucine, and leucine. Five branched-chain aliphatic compounds were identified in these grapes (Supplementary Table S1). Their total concentration was relatively stable in the whole development stages and the highest level was observed at 8 waf. Subsequently, the UV1-A and the UV1-B treated grapes experienced a gradual decreasing trend in the concentration of branched-chain aliphatic volatiles till harvest, whereas the control and the other treated grapes showed a slight increase in the concentration at pre-harvest. As a result, the branched-chain aliphatic volatiles were present at lower concentrations in the UV1-A and the UV1-B treated grapes. Except this, no significant differences were observed between the control and the treated grapes in any other stages (Figure 4). With regards to MPs, only isobutyl methoxypyrazine (IBMP) was detected in the grapes in the early stage of development (Supplementary Table S1). The concentration of IBMP continuously decreased from fruit-set to the onset of veraison, and reached the trace amount afterwards. At 5 waf, the UV1 treatments significantly decreased the concentration of IBMP in the grapes compared to the control (Figure 4). The studies of Gregan *et al.* (2012) [24] and Šuklje *et al*. (2014) [21] also indicated no significant impact of the reduced UV radiation on IBMP concentration in Sauvignon Blanc grapes and wines.

#### 2.1.3. Effect on Fatty Acid-Derived Volatiles

**Figure 5:**
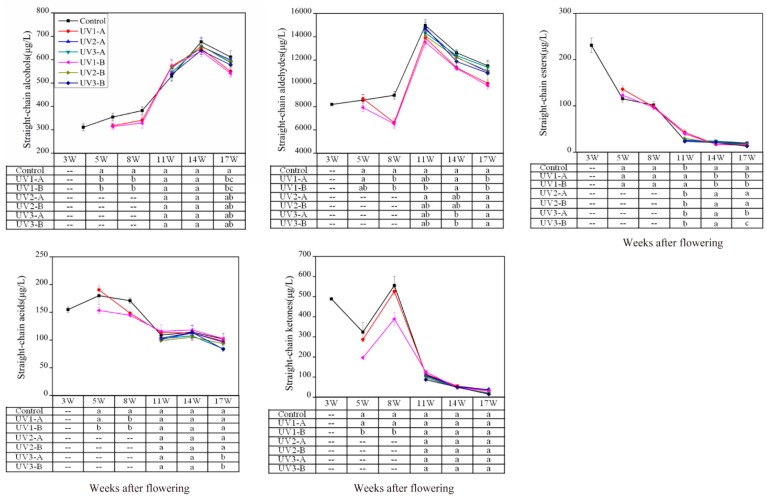
Effect of attenuated UV radiation on the concentration of fatty acid-derived volatiles in developing “Cabernet Sauvignon” grapes. For more information, please refer to the explanation in Figure 2.

Aliphatic volatile compounds include short-chain aldehydes and alcohols that are either straight or branched. They are yielded from fatty acid oxidation and/or amino acid degradation [37]. Straight-chain aliphatic volatiles result from the enzymatic splitting of ployunsaturated fatty acids (mainly linoleic and linolenic acid), and therefore they are also classified as fatty acid-derived volatiles. In grapes, straight-chain alcohols, aldehydes, esters, acids, and ketones belong to straight-chain aliphatic volatiles. In this study, 18 alcohols were identified (Supplementary Table S1). Their total concentration had an increase with grape maturation, and then followed a slight decrease at harvest (Figure 5). The UV1-A and the UV1-B treated grapes had significant lower concentration of straight-chain alcohols compared with the control at 5waf, 8 waf, and at harvest. This indicated that UV radiation attenuation down-regulated the biosynthesis of straight-chain alcohols in Cabernet Sauvignon grapes. No significant differences were observed in the concentrations of alcohols among the UV2 treated, the UV3 treated, and the control grapes.

Straight-chain aldehydes are primary constituents of fatty acid-derived volatiles, and thus determine the evolutionary trend of this group of volatiles. Seventeen aldehydes were detected in the 2010 vintage grapes (Supplementary Table S1). Aldehyde volatiles showed the highest level at 11 waf, followed by a decline at the ripening stage (Figure 5). This was consistent with the report of Kalua *et al*. (2009) [9]. Compared with the control, the UV3 treatments resulted in a decrease in the aldehyde concentrations in the grapes at 14 waf. However, this impact disappeared at harvest (17 waf). The UV1 treatments also induced the reduction of aldehyde concentrations in the whole development period. Particularly at the onset of veraison and at harvest, this reduction was statistically significant (Figure 5). The UV1-B treatment resulted in the grapes with much lower concentration of alcohols and aldehydes compared to the UV1-A treatment (Figure 5), suggesting that the impact of UV radiation reduction could be dose-dependent.

Esters are usually at low levels in grapes with limited flavor contribution. Seven straight-chain esters were identified (Supplementary Table S1). The concentration of ester volatiles showed a gradually decreasing trend. Kalua *et al*. (2009) also reported the similar pattern on the ester volatile evolution, which was mainly explained by the loss of ester synthesis ability in grapes at the beginning of veraison [9]. At pre-harvest, the UV1 and the UV3 treated grapes exhibited a larger reduction in the concentration of straight-chain esters compared with the control (Figure 5). Therefore, the concentration of esters in the UV1 and the UV3 treated grapes were significantly lower than the control at harvest (Figure 5). This suggested that UV radiation attenuation might more rapidly trigger grapes to lose ester synthesis activity at the late development stages of grapes.

Three straight-chain acids were identified from the samples (Supplementary Table S1). Their total concentration decreased overall. In the UV3-A and the UV3-B treated grapes, the concentration of acid volatiles dropped much more than the control, resulting in lower concentration of these compounds at harvest. The UV1 treatments also markedly decreased the concentration of straight-chain acids of the grapes at the early development stages (5 waf and 8 waf) (Figure 5). However, this decrease impact did not remain consistent by berry harvest. Five straight-chain ketones were identified in the 2010 vintage grapes (Supplementary Table S1). The total concentration of these volatiles reached the highest level at 8 waf and subsequently decreased. Apart from the lower concentration in the UV1-B treated grapes in the early development stages (5 waf and 8 waf), no significant differences were observed between the control and the treated grapes (Figure 5).

Lipoxygenase (LOX) is a critical enzyme that has the capacity to convert polyunsaturated fatty acids (PUFAs) into straight-chain aldehydes. These resultant aldehydes can be further reduced to yield alcohols and esters by alcohol dehydrogenase and alcohol acyltransferase, respectively, with the presence of acetic acid [38,39]. LOX, as a defense factor in plants, exerts an important role against complex environments [40]. Gil and his colleagues reported that UV-B exclusion caused a decrease in the concentration of some alcohols, aldehydes, and ketones in Malbec grape berries, which resulted from the alteration of fatty acid metabolisms in plant in response to UV-B exclusion [28]. Kobayashi *et al*. (2011) found that UV radiation reduction delayed the degradation of PUFAs in grapes [41]. These indicated that UV radiation attenuation might down-regulate the metabolism from PUFAs to straight-chain aldehydes, and further to alcohols, acids, ketones, and esters.

### 2.2. Partial Least Square Discriminant Analysis

Based on 83 volatile variables in the grapes at harvest of 2010 vintage, partial least square discriminant analysis (PLS-DA) was applied to sort out individual volatile components that are responsible for differentiation amongst these various treatments. The data were normalized by the Autoscaling method in the MetaboAnalyst 3.0 [42]. The reliability of the discrimination test was evaluated by Leave one out cross-validation (LOOCV) method (Supplementary Figure S1).

**Figure 6:**
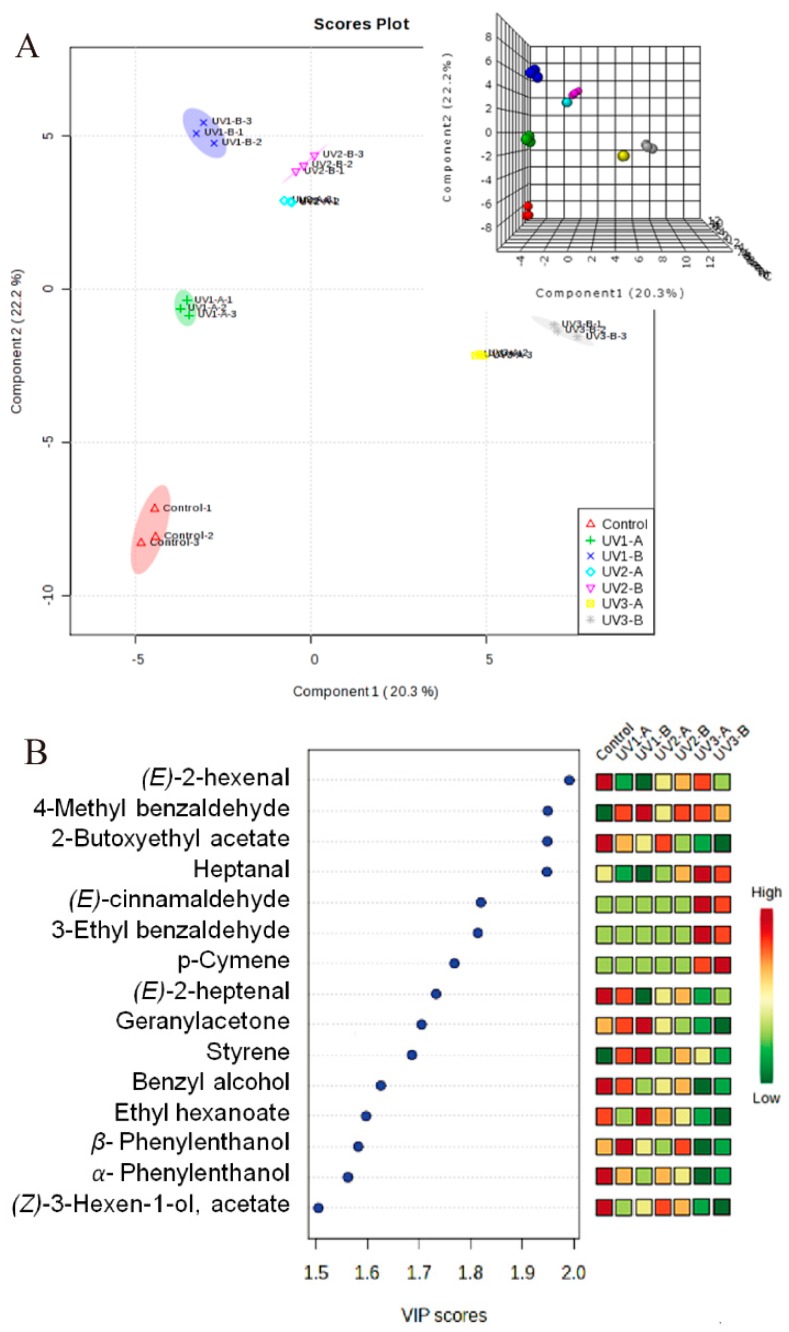
Results of PLS-DA. (**A**) 2D scores plot. The corresponding interactive 3D plot is shown in its top right corner; (**B**) Selected volatile compounds based on VIP scores.

As shown in Figure 6A, these treatments were clearly segregated from each other, and the semi-transparent fields meant 95% confidence intervals. Variables with VIP score >1.5 were considered important contributors for the segregation (Figure 6B). Fifteen major components were screened out, including eight benzenoids, three straight-chain aldehydes, three straight-chain esters, and one C13-norisoprenoid.

Of the selected benzenoids, four benzenoids were detected only in the UV attenuation treated grapes at harvest, contributing to the separation between all the UV attenuation treatments and the control. Of them, *p*-Cymene, 3-ethyl benzaldehyde, and (*E*)-cinnamaldehyde were only present in the UV3-A and the UV3-B treated grapes, whereas 4-methyl benzaldehyde existed in all the UV-attenuation treated grapes but not in the control (Table 1). It is known that the grape maturation stage is crucial for the accumulation of benzene derivatives in grapes [9]. This might explain why the attenuated UV radiation during grape mature period (UV3-A and UV3-B treatments) showed a much more significant promotion on the accumulation of benzenoids. Additionally, the concentration of styrene increased considerably in the grapes with UV attenuation treatments (Figure 6B). About a 3.5-fold enhancement of styrene was observed in the grapes treated by the UV1-A and the UV1-B. On the contrary, both benzyl alcohol and β*-*phenylethanol concentrations were significantly reduced in the UV3-treated grapes. Also, α*-*phenylethanol level was markedly lower in the other UV-treated grape berries except for the UV1-A treatment, and the greatest decrease was found in the UV3-A and the UV3-B treated grapes (Table 1). The alteration of these compounds resulted in the difference in the total concentration of amino acid-derived volatiles between the UV treated grapes and the control (Figure 4). The odor thresholds of benzenoids in grapes tend to be higher than that of other classes of flavor and aroma compounds, like norisoprenoids or methoxypyrazines. From Table 1, it was observed that the concentrations of benzenoid compounds varied from trace levels to hundreds of micrograms per liter, indicating that these chemical species might contribute to the overall odor of juice from these grapes [43].

The attenuated UV radiation around the fruit zone substantially decreased the concentrations of three straight-chain aldehydes in the grapes: (*E*)-2-hexenal, (*E*)-2-heptenal and heptanal. The lowest abundances were all observed in the UV1-B treated grapes (Table 1). These three compounds were found to distinguish the attenuated UV-treated grapes from the control. In grape berries, (*E*)-2-hexenal was the most abundant aldehyde, accounting for 50% of total concentration of straight-chain aldehydes. The influence of UV radiation reduction on this compound eventually caused the difference in aliphatic aldehydes between the UV treated and the control grapes (Figure 5). In grapes, straight-chain aldehydes or alcohols are described as green or leaf-like odors that provide an undesirable flavor perception. Therefore, the attenuated UV radiation might help to eliminate these off flavor attributes.

Both 2-butoxyethyl acetate and (*Z*)-3-hexen-1-ol acetate levels were largely decreased in the grapes treated with the attenuated UV radiation. These two volatiles could be used to distinguish all the UV attenuation treated grapes from the control (Figure 6). Ethyl hexanoate level was also significantly reduced in the UV3 treated grapes (Table 1). The decrease of these volatile concentrations caused the decease of total straight-chain ester level in the attenuated UV radiation treated grapes (Figure 5). In general, esters are less abundant in grapes compared with straight-chain aldehydes and alcohols. They are characterized as fruity that has a pleasant perception. The attenuated UV radiation during grape development is not conducive to improving these positive sensory attributes.

Geranylacetone was the only one component of norisoprenoids that was selected for the differentiation between the attenuated UV treated grapes and the control (Figure 6). The concentration of geranylacetone decreased significantly in the UV3 treated grapes (Table 1). Compared with the other norisoprenoid individuals, geranylacetone showed relatively higher concentration, which determined the evolutionary trend of the total norisoprenoid concentration (Figure 4). Additionally, β-damascenone was not listed as the principal discriminant components. However, it is of importance to grape and wine flavor since this compound has a very low odor detection threshold (0.14 μg/L) with “fruity-flowery”, “honey-like”, and “stewed apple” aromas [44]. In this study, this volatile showed a level above their detection thresholds, and was therefore thought to have an important contribution to the floral boutique. The attenuated UV radiation significantly reduced the concentration of β-damascenone. Particularly, this reduction was the greatest in the UV3-A and the UV3-B treated grapes.

In conclusion, the attenuation of solar UV radiation around grape clusters caused the changes in the accumulation of volatile compounds in developing grape berries, and therefore altered volatile profile. Amino acid-derived volatiles were enhanced by the attenuated UV radiation, such as styrene, 4-methylbenzaldehyde, ethyl benzoate, and benzaldehyde. Fatty acid-derived volatile levels were reduced in response to the attenuated UV radiation, such as (*E*)-2-hexenal and (*E*)-2-heptenal. Moreover, these impacts were depended on development stages. The stage from the onset of veraison to grape harvest turned to be more sensitive for grape berries in respond to attenuated UV radiation (for instance UV3 treatments) regarding volatile profile alteration.

**Table 1:** Concentrations of various volatile compounds in the grapes at harvest in 2010 under field condition (Control) and attenuated UV radiation (μg/L FW).

No.	Class	Calibration Curve	R^2^ Value	Linear Range (μg/L)	Concentration (μg/L FW)						
					Control	UV1-A	UV1-B	UV2-A	UV2-B	UV3-A	UV3-B
Isoprene-derived											
*Terpenes*											
1	β*-*Myrcene	y = 1.572x + 0.0010	0.9885	0.1–3	tr	tr	tr	tr	tr	tr	tr
2	Limonene	y = 4.105x + 0.0012	0.9795	0.1–3	tr	tr	tr	tr	tr	tr	tr
3	Eucalyptol	y = 3.1992x − 0.0003	0.9580	0.1–3	tr	tr	tr	tr	tr	tr	tr
4	γ-Terpinene	y = 3.09x + 0.0017	0.9879	0.1–40	tr	tr	tr	tr	tr	tr	tr
5	Terpinolene	y = 3.8618x + (2 × 10^−5^)	0.9639	0.1–20	tr	tr	tr	tr	tr	tr	tr
6	*cis-*Furan linalool oxide	y = 1.403x − 0.0008	0.9890	0.1–2	tr	tr	tr	tr	tr	tr	tr
7	Dihydro myrcenol	y = 1.7082x + 0.0026	0.9763	0.1–20	0.34 ± 0.03c	tr	0.55 ± 0.03a	0.50 ± 0.03a	tr	0.41 ± 0.05b	0.25 ± 0.03d
8	*trans-*Furan linalool oxide	y = 0.9459x − 0.0028	0.9890	0.1–2	tr	tr	tr	tr	tr	tr	tr
9	Camphor	y = 13.113x − 0.0019	0.9643	0.1–20	2.75 ± 0.32a	2.35 ± 0.28ab	2.10 ± 0.22b	2.60 ± 0.25a	2.50 ± 0.35a	2.93 ± 0.38a	2.53 ± 0.22a
10	Linalool	y = 0.2918x + 0.0036	0.9937	0.1–40	3.87 ± 0.43a	3.92 ± 0.25a	3.83 ± 0.31a	3.72 ± 0.14a	3.74 ± 0.10a	3.82 ± 0.45a	3.76 ± 0.21a
11	Hotrienol	y = 0.433x − 0.0006	0.9971	0.1–40	tr	tr	tr	tr	tr	tr	tr
12	β*-*Cyclocitral	y = 0.0906x + 0.0009	0.9635	0.1–20	0.24 ± 0.03a	0.24 ± 0.02a	tr	0.23 ± 0.03a	tr	0.22 ± 0.02a	0.23 ± 0.01a
13	α-Terpineol	y = 0.1792x + 0.0017	0.9940	0.1–20	1.26 ± 0.12c	2.93 ± 0.13b	4.40 ± 0.35a	4.43 ± 0.25a	3.94 ± 0.50a	3.00 ± 0.21b	3.03 ± 0.23b
14	Borneol	y = 4.0332x + 0.0024	0.9496	0.1–10	2.33 ± 0.22b	2.50 ± 0.22ab	2.87 ± 0.22a	2.95 ± 0.22a	2.53 ± 0.34ab	2.68 ± 0.28a	2.32 ± 0.22b
15	Citral	y = 0.2763x – (8 × 10^−6^)	0.9951	0.1–5	tr	tr	tr	tr	tr	tr	tr
16	β*-*Citronellol	y = 0.4061x + 0.0002	0.9972	0.1–4	tr	tr	tr	tr	tr	tr	tr
17	Geraniol	y = 0.4406x + 0.0075	0.9944	0.3–50	9.07 ± 1.02a	9.02 ± 0.81a	9.13 ± 0.92a	9.10 ± 0.61a	9.09 ± 0.50a	8.75 ± 0.64a	8.83 ± 0.73a
18	α*-*Calacorene	y = 0.375x + 0.0002	0.9795	0.3–20	tr	tr	tr	tr	tr	tr	tr
*Norisoprenoids*											
19	Cyclohexanone, 2,2,6-trimethyl	y = 7.8699x − 0.0021	0.9623	0.1–20	1.22 ± 0.11b	1.36 ± 0.07a	1.47 ± 0.07a	1.34 ± 0.08a	1.35 ± 0.03a	0.77 ± 0.03c	0.69 ± 0.04c
20	5-Hepten-2-one, 6-methyl-	y = 0.2047x + 0.0007	0.9886	0.1–40	1.34 ± 0.28a	1.54 ± 0.13a	1.30 ± 0.16a	1.52 ± 0.22a	1.43 ± 0.10a	1.13 ± 0.05b	1.07 ± 0.01b
21	Dihydroedulan I	y = 0.0893x + 0.0003	0.9958	0.1–20	tr	tr	tr	tr	tr	tr	tr
22	*cis-*Theaspirane	y = 0.2012x + 0.0012	0.9906	0.1–40	tr	tr	tr	tr	tr	tr	tr
23	β*-*Damascenone	y = 0.2377x + (7 × 10^−5^)	0.9952	0.1–10	0.40 ± 0.03a	0.28 ± 0.01bc	0.21 ± 0.01d	0.31 ± 0.02b	0.35 ± 0.03ab	0.26 ± 0.01c	0.26 ± 0.01c
24	Geranylacetone	y = 0.2036x + 0.0007	0.9965	0.1–20	1.92 ± 0.15a	1.95 ± 0.18a	2.25 ± 0.25a	1.85 ± 0.23a	1.81 ± 0.17ab	1.15 ± 0.01b	1.05 ± 0.09b
25	Nerylactone	y = 0.1002x + 0.0023	0.9950	1.0–50	0.80 ± 0.05a	0.90 ± 0.06a	0.75 ± 0.04b	0.76 ± 0.06b	0.82 ± 0.03ab	0.78 ± 0.04b	0.77 ± 0.03b
26	β*-*Ionone	y = 0.1391x + 0.0002	0.9785	0.05–10	0.09 ± 0.01b	tr	tr	tr	tr	0.24 ± 0.01a	0.23 ± 0.01a
Amino acid-derived											
*Benzenoids*											
27	Styrene	y = 3.7779x + 0.0127	0.9850	1–500	53.95 ± 3.36d	182.79 ± 5.25a	200.60 ± 4.12a	99.69 ± 5.12c	140.24 ± 3.52b	133.04 ± 2.15b	96.68 ± 2.25c
28	*p*-Cymene	y = 3.0572x + 0.003	0.9812	0.1–50	tr	tr	tr	tr	tr	2.70 ± 0.12	2.76 ± 0.16
29	Benzaldehyde	y = 0.1682x + 0.0036	0.9907	1–50	5.58 ± 0.10d	10.22 ± 0.82a	10.44 ± 0.21a	7.07 ± 0.43b	9.47 ± 0.21ab	7.50 ± 0.55b	6.61 ± 0.11c
30	4-Methyl benzaldehyde	y = 0.1716x + 0.0003	0.9998	0.1–10	tr	2.98 ± 0.01	2.99 ± 0.01	2.83± 0.01	2.89 ± 0.01	2.86 ± 0.01	2.89 ± 0.01
31	Benzenacetaldehyde	y = 0.321x + 0.0026	0.9786	1–100	16.63 ± 1.23b	19.29 ± 0.82a	20.55 ± 2.22a	19.15 ± 1.21a	19.60 ± 0.56a	16.67 ± 0.34b	16.89 ± 0.78b
32	Acetophenone	y = 0.7011x + 0.0133	0.9814	1–50	13.05 ± 0.97b	15.16 ± 1.01a	15.02 ± 1.17a	13.67 ± 1.11ab	14.44 ± 1.38a	14.24 ± 1.02a	13.90 ± 1.27a
33	Ethyl benzoate	y = 0.0793x + 0.0016	0.9927	0.1–20	tr	1.64 ± 0.25	1.65 ± 0.34	0.81 ± 0.05	1.63 ± 0.03	1.63 ± 0.14	1.62 ± 0.08
34	3-Ethyl benzaldehyde	y = 0.1195x – (7 × 10^−5^)	0.9640	0.1–20	tr	tr	tr	nd	nd	2.77 ± 0.01	2.75 ± 0.01
35	1-(4-Methylphenyl)-ethanone	y = 0.9436x + 0.0075	0.9687	0.1–50	tr	2.61 ± 0.15	2.61 ± 0.10	tr	tr	2.61 ± 0.04	2.60 ± 0.01
36	Naphthalene	y = 7.1162x − 0.0197	0.9849	0.1–100	1.65 ± 0.13a	1.75 ± 0.12a	1.59 ± 0.10a	1.23 ± 0.12c	1.34 ± 0.10ab	1.37 ± 0.10b	1.44 ± 0.10b
37	Methyl salicylate	y = 0.1535x + 0.005	0.9916	0.1–40	15.05 ± 0.60a	15.13 ± 0.42a	15.08 ± 0.21a	15.05 ± 0.29a	15.12 ± 0.37a	15.07 ± 0.23a	15.05 ± 0.21a
38	α-Phenylethanol	y = 4.9179x − 0.0146	0.9984	0.1–40	1.69 ± 0.12a	1.54 ± 0.13a	1.07 ± 0.08c	1.30 ± 0.05b	1.08 ± 0.05c	0.85 ± 0.06d	1.00 ± 0.10cd
39	3,4-Dimethylbenzaldehyde	y = 0.4247x + 0.0027	0.9697	0.1–100	27.31 ± 1.52a	24.93 ± 1.84b	26.01 ± 1.56a	23.12 ± 2.52b	23.49 ± 1.23b	25.12 ± 2.43ab	23.49 ± 1.57b
40	Guaiacol	y = 1.2271x + 0.0008	0.9916	0.05–50	tr	3.17 ± 0.20	6.34 ± 0.14	tr	tr	6.31 ± 0.20	6.28 ± 0.12
41	Benzyl alcohol	y = 16.596x + 0.0065	0.9894	2–500	182.24 ± 9.80a	180.57 ± 10.50a	160.29 ± 10.01ab	161.98 ± 9.48ab	179.23 ± 5.26a	138.28 ± 10.20b	145.29 ± 6.25b
42	2,6-Diterbutyl-4-methyl phenol	y = 0.0922x − 0.0048	0.9612	0.01–8	0.61 ± 0.02b	0.47 ± 0.01c	0.55 ± 0.01b	0.54 ± 0.01b	0.46 ± 0.02c	1.14 ± 0.02a	0.58 ± 0.01b
43	β*-*Phenylethanol	y = 4.8719x − 0.0061	0.9856	10–500	89.03 ± 3.25a	90.14 ± 3.56a	84.84 ± 4.14a	83.84 ± 3.07a	89.65 ± 3.03a	72.03 ± 2.02b	73.87 ± 2.03b
44	4-Methyl phenol	y = 0.5652x + 0.0062	0.9920	0.6–200	16.41 ± 0.59a	16.43 ± 0.66a	16.40 ± 0.42a	16.39 ± 0.41a	16.40 ± 0.51a	16.37 ± 0.41a	16.35 ± 0.35a
45	Phenol	y = 2.1793x − 0.0024	0.9986	0.2–100	23.98 ± 0.52bc	26.58 ± 0.23b	31.30 ± 0.82a	22.49 ± 1.13c	25.81 ± 0.63b	23.96 ± 1.02bc	22.58 ± 0.58c
46	(*E*)-Cinnamaldehyde	y = 0.7087x + 0.012	0.9774	0.1–10	tr	tr	tr	nd	nd	2.65 ± 0.23	2.60 ± 0.16
*Branched-chain aliphatics*											
47	Methyl isobutyl ketone	y = 11.85x − 0.0019	0.9987	0.5–120	616.00 ± 14.26a	571.33 ± 16.20b	559.61 ± 11.06b	630.00 ± 26.55a	620.00 ± 15.15a	600.57 ± 13.25a	600.00 ± 12.15a
48	4,6-Dimethyl-2-heptanone	y = 0.4184x − 0.0004	0.9855	0.1–10	tr	tr	tr	tr	tr	tr	tr
49	3-Methyl-2-buten-1-ol	y = 2.0949x − 0.0003	0.9882	0.05–20	1.41 ± 0.13c	2.65 ± 0.15a	2.48 ± 0.16a	2.10 ± 0.18b	1.93 ± 0.11b	1.80 ± 0.14b	2.55 ± 0.13a
50	1-Hexanol,2-ethyl-	y = 0.073x − 0.0024	0.9987	0.1–26	0.79 ± 0.06b	1.07 ± 0.11a	0.69 ± 0.02c	1.12 ± 0.03a	0.85 ± 0.05b	0.74 ± 0.01b	0.58 ± 0.02c
51	(*S*)-3-ethyl-4-methylpentanol	y = 2.0815x − 0.0003	0.9989	0.4–5	6.75 ± 0.47c	8.33 ± 0.32a	8.70 ± 0.45a	7.12 ± 0.33b	8.25 ± 0.54a	6.34 ± 0.34c	6.17 ± 0.11c
*Methoxypyrazine*											
52	3-Isobutyl-2-methoxypyrazine	y = 106.98x − 0.0012	0.9996	0.1–50	tr	tr	tr	tr	tr	tr	tr
Fatty acid-derived											
*Straight-chain alcohols*											
53	1-Butanol	y = 33.703x − 0.0019	0.9996	0.3–200	24.71 ± 1.20b	25.95 ± 1.31ab	27.26 ± 1.21a	26.60 ± 1.54a	28.66 ± 1.62a	26.79 ± 1.10a	27.26 ± 1.00a
54	(*E*)-2-penten-1-ol	y = 2.6285x − 0.0006	0.9635	0.1–50	10.00 ± 0.82a	9.56 ± 0.46a	9.55 ± 0.56a	9.50 ± 0.34a	9.68 ± 0.72a	9.81 ± 0.61a	9.63 ± 0.50a
55	2-Heptanol	y = 0.3165x − 0.0002	0.9942	0.1–100	1.69 ± 0.11a	1.16 ± 0.07ab	1.04 ± 0.02ab	1.42 ± 0.09a	1.81 ± 0.05a	1.32 ± 0.53	1.51 ± 0.01a
56	(*Z*)-2-penten-1-ol	y=2.9381x − 0.0018	0.9954	1–120	23.89 ± 1.21a	21.26 ± 1.39a	21.52± 1.08a	19.76 ± 1.34b	22.85 ± 1.38a	18.76 ± 1.43b	22.99 ± 1.07a
57	1-Hexanol	y = 0.4184x-0.015	0.9937	0.3–500	481.83 ± 7.24a	432.20 ± 7.12b	418.50 ± 7.56b	470.00 ± 14.97a	468.23 ± 16.66a	467.48 ± 15.30a	464.76 ± 11.77a
58	(*E*)-3-hexen-1-ol	y = 0.3519x + 0.0001	0.9982	0.2–200	21.31 ± 1.20a	20.60 ± 1.04a	21.32 ± 1.10a	22.58 ± 1.14a	21.76 ± 1.24a	20.94 ± 1.85a	21.17 ± 1.02a
59	(*Z*)-3-hexen-1-ol	y = 4.2084x − 0.0042	0.9990	1–500	30.79 ± 2.32b	39.47 ± 1.89a	29.64 ± 2.12b	29.50 ± 1.92b	28.73 ± 2.51b	31.23 ± 3.13b	33.23 ± 3.68b
60	(*E*)-2-hexen-1-ol	y = 2.1862x − 0.0605	0.9627	1–500	29.19 ± 2.70a	20.67 ± 0.88b	20.14 ± 1.88b	22.00 ± 1.56b	24.00 ± 2.70b	24.41 ± 1.23b	25.48 ± 1.23b
61	(*Z*)-2-hexen-1-ol	y = 3.0267x − 0.0027	0.9983	0.3–200	5.34 ± 0.84bc	2.47 ± 0.12d	2.38 ± 0.22d	9.02 ± 0.58a	6.35 ± 0.23b	3.94 ± 0.38c	4.43 ± 0.35bc
62	2-Octanol	y = 0.1804x + 0.0002	0.9947	0.1–120	0.26 ± 0.01a	0.25 ± 0.01a	0.23 ± 0.01a	0.28 ± 0.02a	0.27 ± 0.01a	0.27 ± 0.01a	0.29 ± 0.01a
63	1-Octen-3-ol	y = 0.1343x-0.0004	0.9967	0.1–50	1.55 ± 0.12a	1.47 ± 0.10b	1.27 ± 0.08b	1.55 ± 0.06a	1.62 ± 0.13a	1.36 ± 0.12ab	1.75 ± 0.15a
64	1-Heptanol	y = 0.2501x − 0.0001	0.9888	0.04–300	0.21 ± 0.01b	0.20 ± 0.01b	0.18 ± 0.01b	0.30 ± 0.02a	0.26 ± 0.02a	0.20 ± 0.01b	0.29 ± 0.01a
65	2-Nonanol	y = 0.047x + 0.0008	0.9985	0.1–100	0.87 ± 0.06a	0.84 ± 0.05a	0.84 ± 0.05a	0.86 ± 0.01a	0.87 ± 0.02a	0.85 ± 0.02a	0.86 ± 0.02a
66	1-Octanol	y = 0.2199x + 0.0003	0.9980	0.3–200	0.63 ± 0.05b	0.75 ± 0.02a	0.64 ± 0.02b	0.74 ± 0.12a	0.70 ± 0.04ab	0.62 ± 0.05b	0.74 ± 0.06a
67	(*E*)*-*2-octen-1-ol	y = 0.1313x − 0.0002	0.9885	0.05–10	0.42 ± 0.02b	0.35 ± 0.02cd	0.33 ± 0.02d	0.41 ± 0.01b	0.43 ± 0.01b	0.37 ± 0.03c	0.47 ± 0.01a
68	1-Nonanol	y = 0.0572x + 0.0002	0.9857	0.1–20	0.85 ± 0.03a	0.84 ± 0.02a	0.83 ± 0.04a	0.85 ± 0.08a	0.84 ± 0.02a	0.83 ± 0.05a	0.84 ± 0.05a
69	1-Decanol	y = 0.2717x + 0.0045	0.9950	0.1–20	0.98 ± 0.05a	1.00 ± 0.14a	1.12 ± 0.11a	0.95 ± 0.06a	1.05 ± 0.08a	1.08 ± 0.04a	0.97± 0.04a
70	1-Dodecanol	y = 0.4996x + 0.0018	0.9987	0.1–100	1.99 ± 0.10a	2.06 ± 0.02a	2.00 ± 0.04a	2.13 ± 0.25a	1.96 ± 0.04a	2.28 ± 0.22a	1.97 ± 0.05a
*Straight-chain aldehydes*											
71	Butanal	y = 5.9567x + 0.043	0.9983	1–200	61.83 ± 2.51a	61.51 ± 2.13a	59.35 ± 3.95a	60.36 ± 2.32a	60.50 ± 2.00a	56.57 ± 4.03a	53.15 ± 2.25b
72	Pentanal	y = 5.5978x + 0.1834	0.9668	10–500	56.14 ± 2.26a	56.73 ± 1.56a	56.39 ± 2.13a	56.33 ± 2.23a	56.02 ± 2.55a	53.05 ± 2.13b	50.55 ± 1.87b
73	Hexanal	y = 5.7615x − 0.1338	0.9999	100–5000	4625.22 ± 50.20a	4218.57 ± 78.30b	4199.11 ± 85.30b	4578.71 ± 64.00a	4727.43 ± 155.20a	4500.00 ± 82.30a	4600.00 ± 73.20a
74	(*Z*)-3-hexenal	y = 5.095x + 0.0644	0.9958	5–300	168.85 ± 4.68a	155.88 ± 8.82a	142.83 ± 5.70b	150.00 ± 3.60b	159.88 ± 5.30a	136.50 ± 5.60c	118.79 ± 4.53d
75	Heptanal	y = 3.3543x − 0.0042	0.9908	4–200	22.51 ± 1.02a	14.49 ± 0.89b	10.99 ± 0.85b	21.27 ± 1.23a	23.00 ± 0.98a	24.11 ± 1.78a	23.56 ± 0.56a
76	(*E*)-2-hexenal	y = 5.915x − 0.5077	0.9994	650–20000	6000.00 ± 83.20a	5275.21 ± 65.30c	5174.30 ± 64.30c	5399.94 ± 95.30b	5496 ± 62.30b	5838.32 ± 184.30a	5299.82 ± 72.50c
77	Octanal	y = 5.0692x − 0.0017	0.9839	0.1–20	4.07 ± 0.12a	3.59 ± 0.20b	4.13 ± 0.08a	3.36 ± 0.15c	3.52 ± 0.10b	3.75 ± 0.10b	2.99 ± 0.10d
78	(*E*)-2-heptenal	y = 0.3201x − 0.0002	0.9984	0.1–10	0.48 ± 0.04a	0.38 ± 0.02b	0.25± 0.01d	0.37± 0.01b	0.37 ± 0.03b	0.28 ± 0.02cd	0.32 ± 0.03c
79	Nonanal	y = 1.1837x − 0.0042	0.9862	0.1–100	2.86 ± 0.16a	1.15 ± 0.11c	3.06 ± 0.30a	2.50 ± 0.19b	2.59 ± 0.15b	3.04 ± 0.13a	2.56 ± 0.13b
80	(*E*,*E*)-2,4-hexadienal	y = 4.8522x + 0.2187	0.9837	5–1000	158.97 ± 7.58a	146.65 ± 11.37a	139.37 ± 4.60b	154.21 ± 5.30a	162.13 ± 4.50a	124.47 ± 3.40c	112.78 ± 2.30d
81	(*E*)-2-octenal	y = 0.1313x + 0.0007	0.9883	0.1–20	0.42 ± 0.03a	0.37 ± 0.02ab	0.37 ± 0.03ab	0.36 ± 0.02b	0.48 ± 0.04a	0.35 ± 0.02b	0.35 ± 0.03b
82	(*E*,*E*)-2,4-heptadienal	y = 0.5649x − 0.0015	0.9979	1–300	85.50 ± 5.40a	81.39 ± 1.10a	81.40 ± 1.79a	81.49 ± 2.80a	79.01 ± 3.56a	83.97 ± 5.23a	83.18 ± 3.53a
83	Decanal	y = 3.5466x − 0.0206	0.9967	0.1–100	6.37 ± 0.16a	4.84 ± 0.23bc	4.44 ± 0.11c	5.38 ± 0.13b	4.26 ± 0.23c	3.91 ± 0.19c	3.81 ± 0.21c
84	(*E*)-2-nonenal	y = 0.0709x – (4 × 10^−5^)	0.9881	0.01–10	0.69 ± 0.04a	0.74 ± 0.05a	0.69 ± 0.02a	0.64 ± 0.01a	0.68 ± 0.01a	0.68 ± 0.01a	0.63 ± 0.05a
85	(*E*,*Z*)-2,6-nonadienal	y = 0.0462x + 0.0009	0.9937	0.1–4	0.35 ± 0.01b	0.32 ± 0.01c	0.36 ± 0.02b	0.35 ± 0.02b	0.28 ± 0.02d	0.44 ± 0.04a	0.38 ± 0.03a
86	(*E*,*E*)-2,4-nonadienal	y = 0.0572x + 0.0002	0.9857	0.1–4	0.34 ± 0.01a	0.30 ± 0.03ab	0.25 ± 0.01c	0.34 ± 0.01a	0.32 ± 0.01a	0.28 ± 0.02b	0.23 ± 0.01c
87	Dodecanal	y = 0.0353x – (4 × 10^−5^)	0.9867	0.1–5	0.49 ± 0.03b	0.57 ± 0.04a	0.55 ± 0.03a	0.42 ± 0.01c	0.40 ± 0.02c	0.45 ± 0.03b	0.46 ± 0.02b
*Straight-chain esters*											
88	Ethyl acetate	y = 3.3663x + 0.1055	0.9959	1–60	4.21 ± 0.20b	4.66 ± 0.25b	6.39 ± 0.10a	4.81 ± 0.20b	6.86 ± 0.50a	6.57 ± 0.30a	6.20 ± 0.48a
89	Ethyl butanoate	y = 0.5709x – (8 × 10^−5^)	0.9811	0.1–5	tr	tr	tr	tr	tr	tr	tr
90	2-Butoxyethyl acetate	y = 1.1038x − 0.0008	0.9959	0.1–20	3.25 ± 0.08a	1.37 ± 0.10b	1.04 ± 0.06bc	1.54 ± 0.07b	1.04 ± 0.08c	0.89 ± 0.03d	0.40 ± 0.01e
91	Hexyl acetate	y = 0.5956x + 0.0037	0.9867	1–50	4.17 ± 0.10a	4.52 ± 0.25a	4.76 ± 0.18a	4.70 ± 0.31a	4.63 ± 0.15a	3.91 ± 0.17a	3.82 ± 0.03a
92	Ethyl hexanoate	y = 2.1749x – 0.1021	0.9956	0.1–30	1.59 ± 0.15a	1.42 ± 0.07a	1.64 ± 0.05a	1.55 ± 0.11a	1.54 ± 0.15a	1.13 ± 0.07b	1.03 ± 0.09b
93	(*Z*)-3-hexen-1-ol, acetate	y = 5.1939x − 0.0037	0.9980	0.1–100	5.37 ± 0.19a	1.16 ± 0.11e	1.64 ± 0.04d	4.32 ± 0.29b	2.45 ± 0.10c	0.90 ± 0.11e	0.33 ± 0.05f
94	Ethyl octanoate	y = 0.6077x + 0.0006	0.9899	0.5–50	1.21 ± 0.04a	1.17 ± 0.09a	1.27 ± 0.10a	1.24 ± 0.09a	1.13 ± 0.23a	0.96 ± 0.01a	1.00 ± 0.09a
*Straight-chain acids*											
95	Hexanoic acid	y = 2.7019x + 0.0793	0.9931	1–700	101.93 ± 10.11a	98.18 ± 2.21a	102.63 ± 3.21a	96.32 ± 2.13a	93.52 ± 3.25a	84.33 ± 2.12b	83.57 ± 3.13b
96	(*E*)-3-Hexenoic acid	y = 5.1482x − 0.0028	0.9976	0.1–5	tr	tr	tr	tr	tr	tr	tr
97	Nonanoic acid	y = 0.3257x − 0.0066	0.9884	0.1–5	tr	tr	tr	tr	tr	tr	tr
*Straight-chain ketones*											
98	1-Penten-3-one	y = 12.315x − 0.0004	0.9685	0.1–120	29.95 ± 1.35a	27.00 ± 1.85a	28.00 ± 1.35a	33.30 ± 2.68	27.73 ± 2.12	30.00 ± 2.38	28.60 ± 2.02
99	3-Octanone	y = 0.0725x – (2 × 10^−5^)	0.9608	0.1–10	0.91 ± 0.06b	1.00 ± 0.04b	1.58 ± 0.08a	1.03 ± 0.05b	0.86 ± 0.06b	1.23 ± 0.10b	1.07 ± 0.07b
100	2-Octanone	y = 4.7791x − 0.0019	0.8967	0.1–20	1.65 ± 0.12b	1.80 ± 0.14b	1.63 ± 0.15b	1.55 ± 0.10b	1.39 ± 0.08c	2.50 ± 0.13a	1.68 ± 0.12b
101	1-Octen-3-one	y = 2.008x + 0.0032	0.9058	0.1–40	3.47 ± 0.32ab	3.69 ± 0.30ab	4.24 ± 0.53a	3.27 ± 0.32b	3.13 ± 0.28b	3.48 ± 0.33ab	3.75 ± 0.25ab
102	2,3-Octanedione	y = 3.355x-0.4503	0.9873	0.1–5	0.50 ± 0.04a	0.53 ± 0.05a	0.45 ± 0.05a	0.55 ± 0.05a	0.42 ± 0.08b	0.52 ± 0.05a	0.48 ± 0.05a

nd: not detected; tr: trace level. There are three kinds of attenuated UV radiation treatments with polyester film A and film B: UV1-A/B: solar UV is excluded from 3-week after flowering to grape harvest; UV2-A/B: from 3-week after flowering to the onset of veraison; UV3-A/B: from the onset of veraison to grape harvest. Different letters represent significant variance among the control and treatments at the 0.05 level according to the One-way ANOVA test.

## 3. Experimental Section

### 3.1. Plant Materials

This study was conducted using 2009 and 2010 vintages in a commercial vineyard in Huailai, Hebei province, China (latitude 40°N, longitude 115°E). The own-rooted vines (*Vitis vinifera* L. cv. Cabernet Sauvignon) were planted in the year 2000 in north-south row orientation with row spacing of 2.5 m × 1.0 m. The vines were managed according to industry standards for irrigation, nutrition, and disease/pest management. In this vineyard, the vines were trained to form vertical trunk with horizontal cordon at 0.8 m above ground and being spur-pruned.

### 3.2. Field Treatments and Sampling

**Figure 7:**
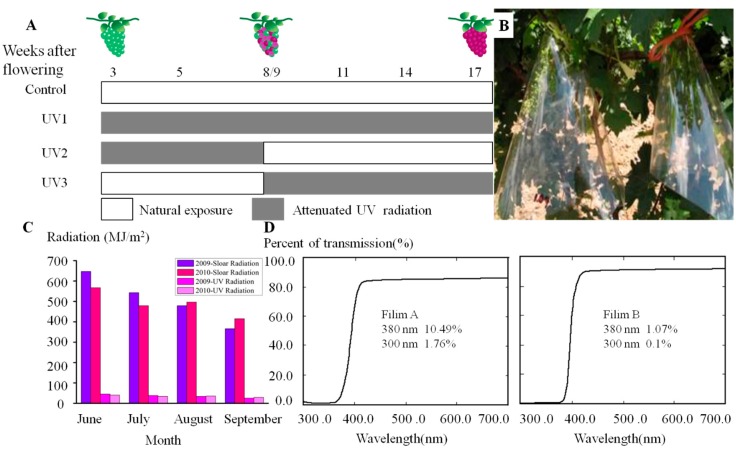
(**A**) Experimental design of the attenuated UV radiation treatments in the field. The control indicates that grape bunch is naturally exposed to sunlight. UV1 represents that UV radiation reaching grape bunch surface is artificially attenuated from fruit-set (3waf) to harvest (17waf); UV2 from fruit-set to the onset of veraison (8 waf in the 2010 vintage and 9 waf in 2009); UV3 from the onset of veraison to harvest; (**B**) Field photography of the attenuated UV radiation via polyester film; (**C**) Monthly total solar radiation and UV radiation in the experimental region during June to September in the two vintages; (**D**) Spectral information of polyester film A and film B. The data shown within two plots are the transmittance of solar light with wavelengths of less than 380 nm and 300 nm, respectively.

Three attenuated UV treatments, together with the control, were carried out. Polyester films A and B (Luckyfilm Co., Ltd., Baoding, China) were used to absorb solar UV components. A total of 60 grapevines were chosen for three replicates with each consisting of 20 plots planted with single trees. During the experiments, each grapevine contained a main vine with 14–15 fruiting branches. On each vine, two or three bunches were naturally exposed throughout the whole development (the control), and others were wrapped with film A or film B at specific developmental stages (treatments). As shown in Figure 7A, there were three types of attenuated UV radiation treatments: UV1 (polyester film was applied from 3 weeks after flowering (waf) to harvest), UV2 (from 3 waf to onset of veraison), and UV3 (from the onset of veraison to harvest). Film was rolled into an inverted funnel shape, opening on both ends, to assure similar temperature and humidity inside and outside. The polyester film was fixed on a fruit stem with a colored rope. Film A cut off 89.51% invisible sunlight of wavelengths below 380 nm (including majority of UV-A and total UV-B), whereas film B removed 98.97% invisible sunlight (including almost all UV-A and UV-B irradiation) correspondingly (Figure 7B).

In the year of 2009, we applied the UV1 treatments with film A and film B with the objective of assessing the effect of attenuated UV radiation throughout grape development on grape volatile profiling. In 2010, three treatments (UV1, UV2 and UV3) with film A or film B were carried out to assess whether the effect of attenuated UV radiation was depended on development stages. Monthly solar radiation and UV radiation during fruit growing season in both of years, were provided by the China Meteorological Data Sharing Service System. From Figure 7B, we found that monthly solar radiation declined from June to August but UV radiation raised by approximately 7.2% of solar radiation in this region. There were no large differences in monthly UV radiation between both of vintages. For each treatment about 100–150 fruits per replicate were collected from different vines and bunches. Sampling was done at about 10 am at the indicated date. In the present study, veraison began at 9 waf in 2009 and at 8 waf in 2010, respectively, and fruit harvest was at 17 waf in both of vintages. The samples were transported to the laboratory on ice and immediately frozen in liquid nitrogen prior to storage at −80 °C.

### 3.3. Extraction of Volatile Compounds

Sample preparation included grinding and cold stabilization according to our previous report [45]. Seeds were removed from berries. The remaining parts (100 g) were ground into powder by using a 20,000 rpm grinder under an addition of 1 g polyvinylpolypyrrolidone (PVPP) and the protection of liquid nitrogen. After being macerated for 120 min at 4 °C, the juice was centrifuged at 6000× *g* for 10 min. The clear juice (5 mL) was transferred to a 15 mL vial and blended with 1 g of NaCl and 10 μL of internal standard 4-methyl-2-pentanol (4M2P, 1.0018 g/L). The vial was tightly capped with a PTFE-silicon septum containing a magnetic stirrer. Afterwards, the vial containing the sample was equilibrated at 40 °C for 30 min with stirring (300 rpm). A SPME fibre coated with 50/30 μm divinylbenzene/carboxen/polydimethylsiloxane (DVB/CAR/PDMS; Supelco, Bellefonte, PA, USA) was then inserted into the headspace and conducted the extraction for 30 min with continuous agitation and heating. The fibre was subsequently desorbed in the GC injector for 8 min at 250 °C. For each biological replicate sample, two technological replicates were carried out.

### 3.4. GC-MS Analysis

GC-MS was used to analyze volatile compounds according to our previous method [45]. The GC-MS system was an Agilent 6890 GC equipped with a HP-INNOWAX capillary column (60 m × 0.25 mm × 0.25 μm; J & W Scientific, San Francisco, CA, USA), coupled with an Agilent 5975 mass spectrometry (Agilent Technologies, Inc., Santa Clara, CA, USA). The helium carrier gas was at a flow rate of 1 mL/min. The column temperature program was as follows: holding 50 °C for 1 min, increasing at 3.0 °C/min to 220 °C and holding 220 °C for 5 min. The temperature of interface of GC and MS was set at 280 °C. The ion source temperature was 230 °C. The electron impact (EI) at 70 eV was scanned in the range of *m*/*z* 30–350.

### 3.5. Qualitative and Quantitative Analysis

Retention indices were calculated after analyzing C6-C24 *n*-alkane series (Supelco, Bellefonte, PA, USA) under the same chromatographic conditions. Identifications were based on mass spectra matching in the standard NIST11 library and retention indices of reference standards in authors’ laboratories. For compound quantitation, a synthetic matrix was prepared in distilled water containing 200 g/L glucose and 7 g/L tartaric acid, and the pH was adjusted to 3.3 using 5 M NaOH solution. Standards were dissolved with ethanol (HPLC quality) and diluted into ten levels in succession with the synthetic matrix. Volatile compounds of each level were extracted and analyzed using the same method as the grape samples. The peak areas calculated by ChemStation software (Agilent Technologies, Inc.) on selective ion mode (SIM) were used for quantification. The calibration curve of each compound was established by the ratio of area ratio against concentration. And concentrations of volatile compounds were calculated through their calibration curves. For the compounds that lacked chemical standards, their concentration were integrated using the standards that possessed the same functional group and/or similar numbers of C atoms.

### 3.6. Statistical Analysis

A one-way analysis of variance (ANOVA) was used to measure differences in the concentrations of volatiles among the treated groups and the control, employing Duncan’s multiple range tests at a level of *p* < 0.05 using SPSS version 20.0 statistical package for windows (SPSS Inc., Chicago, IL, USA). PLS-DA, Partial Least Square Discriminant Analysis was performed by Web-based tool Metabo-Analyst 3.0 and auto-scaling (mean-centered and divided by the standard deviation of each variable) was used in normalization procedure [42]. Origin 8.0 (Origin Lab Inc., Northampton, MA, USA) was used to draft the graph. Each data point, expressed as milligram equivalent of the respective standard per liter of grape juice, was the average of three replications, *n* = 3.

## Supplementary Materials

Supplementary materials can be accessed at: http://www.mdpi.com/1420-3049/20/09/16946/s1.
