# Supplementary material for: Attenuated UV Radiation Alters Volatile Profile in Cabernet Sauvignon Grapes under Field Conditions
Source: Molecules. 2015 Sep 17;20(9):16946–69. doi: 10.3390/molecules200916946 (PMC6331812; doi:10.3390/molecules200916946)
Supplement: Supplementary file 1 [file molecules-20-16946-s001.pdf]

Supplementary Materials

Table S1. Volatile compounds identified in the grapes of 2009 and 2010 vintages in this study.

Classes	Compounds	RI [a]	LRI [b]	2009			2010						
				Control	UV1-A	UV1-B	Control	UV1-A	UV1-B	UV2-A	UV2-B	UV3-A	UV3-B
Isoprene-derived													
Terpenes													
	β-Myrcene	1164	1174	√	√	√	√	√	√	√	√	√	√
	Limonene	1185	1180	√	√	√	√	√	√	√	√	√	√
	Eucalyptol	1198	1210	√	√	√	√	√	√	√	√	√	√
	γ-Terpinene	1230	1238	--	--	--	√	√	√	√	√	√	√
	Terpinolene	1270	1274	√	√	√	√	√	√	√	√	√	√
	cis-Furan linalool oxide	1439	1478	√	√	√	√	√	√	√	√	√	√
	Dihydro myrcenol	1473	1473	--	--	--	√	√	√	√	√	√	√
	trans-Furan linalool oxide	1468	1452	--	--	--	√	√	√	√	√	√	√
	Camphor	1520	1532	√	√	√	√	√	√	√	√	√	√
	Linalool	1532	1553	√	√	√	√	√	√	√	√	√	√
	Hotrienol	1610	1614	--	--	--	√	√	√	√	√	√	√
	β-Cyclocitral	1628	1600	√	√	√	√	√	√	√	√	√	√
	α-Terpineol	1701	1707	√	√	√	√	√	√	√	√	√	√
	Borneol	1710	1719	--	--	--	√	√	√	√	√	√	√
	Citral	1726	1733	--	--	--	√	√	√	√	√	√	√
	β-Citronellol	1741	1772	--	--	--	√	√	√	√	√	√	√
	Geraniol	1853	1857	√	√	√	√	√	√	√	√	√	√
	α-Calacorene	1916	1914	--	--	--	√	√	√	√	√	√	√
Norisoprenoids													
	Cyclohexanone, 2,2,6-trimethyl	1282	1328	--	--	--	√	√	√	√	√	√	√
	5-Hepten-2-one,6-methyl	1336	1348	√	√	√	√	√	√	√	√	√	√
	Dihydroedulan I	1342	1296	--	--	--	√	√	√	√	√	√	√
	cis-Theaspirane	1520	1553	√	√	√	√	√	√	√	√	√	√

Table S1. *Cont.*

Classes	Compounds	RI [a]	LRI [b]	2009					2010				
				Control	UV1-A	Control	UV1-A	Control	UV1-A	Control	UV1-A	Control	UV1-A
	β-Damascenone	1833	1838	√	√	√	√	√	√	√	√	√	√
	Geranylacetone	1864	1868	√	√	√	√	√	√	√	√	√	√
	Nerylactone	1866	1865	√	√	√	√	√	√	√	√	√	√
	β-Ionone	1952	1964	√	√	√	√	√	√	√	√	√	√
Amino acid-derived													
Benzenoids													
	Styrene	1255	1248	√	√	√	√	√	√	√	√	√	√
	p-Cymene	1295	1280	--	--	--	√	√	√	√	√	√	√
	Benzaldehyde	1532	1541	√	√	√	√	√	√	√	√	√	√
	4-Methyl benzaldehyde	1630	1642	√	√	√	√	√	√	√	√	√	√
	Benzenacetaldehyde	1644	1663	√	√	√	√	√	√	√	√	√	√
	Acetophenone	1656	1662	--	--	--	√	√	√	√	√	√	√
	Ethyl benzoate	1676	1681	--	--	--	√	√	√	√	√	√	√
	3-Ethyl benzaldehyde	1680	1168[c]	--	--	--	√	√	√	--	--	√	√
	1-(4-Methylphenyl)-ethanone	1750	1797	--	--	--	√	√	√	√	√	√	√
	Naphthalene	1756	1765	√	√	√	√	√	√	√	√	√	√
	Methyl salicylate	1792	1798	--	--	--	√	√	√	√	√	√	√
	α-Phenylethanol	1812	1816	√	√	√	√	√	√	√	√	√	√
	3,4-Dimethylbenzaldehyde	1829	1790	√	√	√	√	√	√	√	√	√	√
	Guaiacol	1862	1859	--	--	--	√	√	√	√	√	√	√
	Benzyl alcohol	1892	1872	√	√	√	√	√	√	√	√	√	√
	2,6-Diterbutyl-4-methyl phenol	1910	1910	√	√	√	√	√	√	√	√	√	√
	β-Phenylethanol	1928	1937	√	√	√	√	√	√	√	√	√	√
	4-Methyl phenol	2010	2031	√	√	√	√	√	√	√	√	√	√
	Phenol	2029	2014	√	√	√	√	√	√	√	√	√	√
	(*E*)-Cinnamaldehyde	2040	2043	--	--	--	√	√	√	--	--	√	√

Table S1. *Cont.*

Classes	Compounds	RI [a]	LRI [b]		2009					2010			
				Control	UV1-A	Control	UV1-A	Control	UV1-A	Control	UV1-A	Control	UV1-A
Branched-chain aliphatics													
	Methyl isobutyl ketone	1007	1002	--	--	--	√	√	√	√	√	√	√
	4,6-Dimethyl-2-heptanone	1262	1262	√	√	√	√	√	√	√	√	√	√
	3-Methyl-2-buten-1-ol	1302	1295	√	√	√	√	√	√	√	√	√	√
	1-Hexanol,2-ethyl	1465	1476	√	√	√	√	√	√	√	√	√	√
	(*S*)-3-ethyl-4-ethylpentanol	1506	1506	√	√	√	√	√	√	√	√	√	√
	Octanoic acid, 3-methylbutyl ester	1645	1654	√	√	√	--	--	--	--	--	--	--
Methoxypyrazine													
	3-Isobutyl-2-methoxypyrazine	1525	1520	√	√	√	√	√	√	--	--	--	--
Fatty acid-derived													
Straight-chain alcohols													
	1-Butanol	1158	1155	√	√	√	√	√	√	√	√	√	√
	(*E*)-2-penten-1-ol	1309	1313	√	√	√	√	√	√	√	√	√	√
	2-Heptanol	1311	1320	√	√	√	√	√	√	√	√	√	√
	(*Z*)-2-penten-1-ol	1324	1323	--	--	--	√	√	√	√	√	√	√
	1-Hexanol	1344	1361	√	√	√	√	√	√	√	√	√	√
	(*E*)-3-hexen-1-ol	1356	1358	√	√	√	√	√	√	√	√	√	√
	(*Z*)-3-hexen-1-ol	1378	1392	√	√	√	√	√	√	√	√	√	√
	(*E*)-2-hexen-1-ol	1400	1405	√	√	√	√	√	√	√	√	√	√
	(*Z*)-2-hexen-1-ol	1410	1403	√	√	√	√	√	√	√	√	√	√
	2-Octanol	1416	1421	√	√	√	√	√	√	√	√	√	√
	1-Octen-3-ol	1443	1452	√	√	√	√	√	√	√	√	√	√
	1-Heptanol	1455	1457	√	√	√	√	√	√	√	√	√	√
	2-Nonanol	1513	1525	√	√	√	√	√	√	√	√	√	√
	1-Octanol	1554	1562	√	√	√	√	√	√	√	√	√	√
	(*E*)-2-octen-1-ol	1614	1615	√	√	√	√	√	√	√	√	√	√

Table S1. *Cont.*

Classes	Compounds	RI [a]	LRI [b]	2009			2010						
				Control	UV1-A	Control	UV1-A	Control	UV1-A	Control	UV1-A	Control	UV1-A
	1-Nonanol	1664	1658	√	√	√	√	√	√	√	√	√	√
	1-Decanol	1765	1766	--	--	--	√	√	√	√	√	√	√
	1-Dodecanol	1950	1973	--	--	--	√	√	√	√	√	√	√
Straight-chain aldehydes													
	Butanal	875	877	--	--	--	√	√	√	√	√	√	√
	Pentanal	982	993	√	√	√	√	√	√	√	√	√	√
	Hexanal	1099	1079	√	√	√	√	√	√	√	√	√	√
	(*Z*)-3-hexenal	1146	1135	√	√	√	√	√	√	√	√	√	√
	Heptanal	1175	1186	√	√	√	√	√	√	√	√	√	√
	(*E*)-2-hexenal	1230	1209	√	√	√	√	√	√	√	√	√	√
	Octanal	1283	1296	√	√	√	√	√	√	√	√	√	√
	(*E*)-2-heptenal	1323	1315	√	√	√	√	√	√	√	√	√	√
	Nonanal	1389	1401	√	√	√	√	√	√	√	√	√	√
	(*E*,*E*)-2,4-hexadienal	1402	1392	√	√	√	√	√	√	√	√	√	√
	(*E*)-2-octenal	1430	1441	√	√	√	√	√	√	√	√	√	√
	(*E*,*E*)-2,4-heptadienal	1480	1462	√	√	√	√	√	√	√	√	√	√
	Decanal	1500	1506	√	√	√	√	√	√	√	√	√	√
	(*E*)-2-nonenal	1537	1525	√	√	√	√	√	√	√	√	√	√
	(*E*,*Z*)-2,6-nonadienal	1575	1599	√	√	√	√	√	√	√	√	√	√
	(*E*,*E*)-2,4-nonadienal	1660	1705	--	--	--	√	√	√	√	√	√	√
	Dodecanal	1690	1722	--	--	--	√	√	√	√	√	√	√
Straight-chain esters													
	Ethyl acetate	877	888	√	√	√	√	√	√	√	√	√	√
	Ethyl butanoate	1047	1033	√	√	√	√	√	√	√	√	√	√
	2-Butoxyethyl acetate	1090	1053	--	--	--	√	√	√	√	√	√	√
	Hexyl acetate	1228	1282	√	√	√	√	√	√	√	√	√	√
	Ethyl hexanoate	1260	1250	√	√	√	√	√	√	√	√	√	√

Table S1. *Cont.*

Classes	Compounds	RI [a]	LRI [b]	2009					2010				
				Control	UV1-A	Control	UV1-A	Control	UV1-A	Control	UV1-A	Control	UV1-A
	(*Z*)-3-hexen-1-ol, acetate	1311	1324	√	√	√	√	√	√	√	√	√	√
	Ethyl octanoate	1429	1421	√	√	√	√	√	√	√	√	√	√
Straight-chain acids													
	Hexanoic acid	1860	1866	√	√	√	√	√	√	√	√	√	√
	(*E*)-3-Hexenoic acid	1950	1930	--	--	--	√	√	√	√	√	√	√
	Nonanoic acid	2190	2175	--	--	--	√	√	√	√	√	√	√
Straight-chain ketones													
	1-Penten-3-one	1010	1017	--	--	--	√	√	√	√	√	√	√
	3-Octanone	1250	1265	√	√	√	√	√	√	√	√	√	√
	2-Octanone	1290	1284	--	--	--	√	√	√	√	√	√	√
	1-Octen-3-one	1305	1310	√	√	√	√	√	√	√	√	√	√
	2,3-Octanedione	1345	1360	--	--	--	√	√	√	√	√	√	√

[a] Retention indices on HP-Innowax column. [b] Linear retention indices on standard polar column referenced from NIST 11 database. [c] Linear retention index on semi-polar column referenced from NIST 11 database. √ Means detected, -- means undetected.

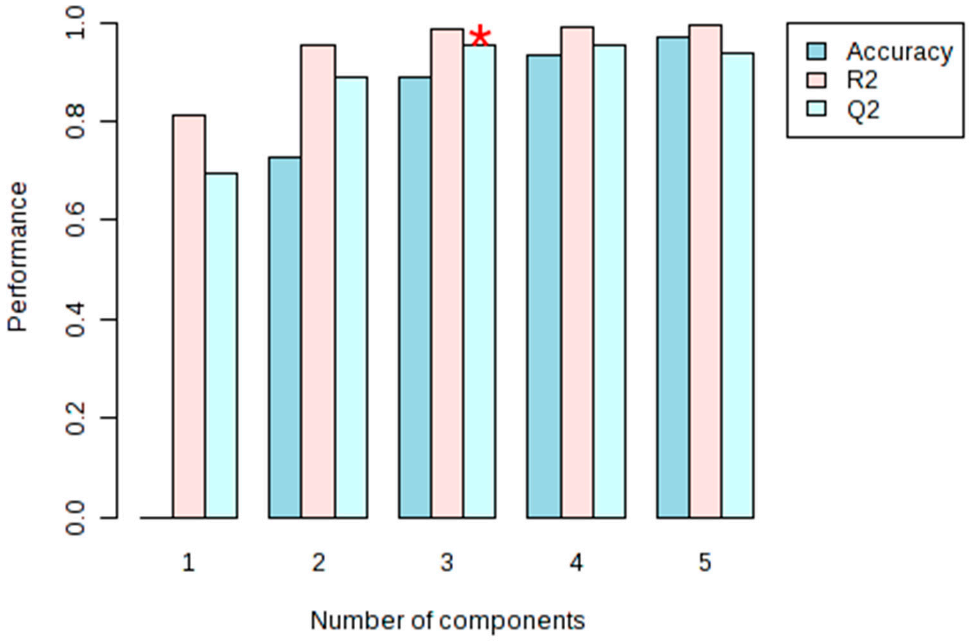

Figure S1. R^2 and Q^2 of PLS-DA by LOOCV method. * Grapes under various treatments were clearly separated by the first three principle components.
